# RACK1 Promotes Meningioma Progression by Activation of NF-κB Pathway via Preventing CSNK2B from Ubiquitination Degradation

**DOI:** 10.3390/cancers16040767

**Published:** 2024-02-13

**Authors:** Ali Abdi Maalim, Zihan Wang, Yimin Huang, Ting Lei

**Affiliations:** 1 Department of Neurosurgery, Tongji Hospital of Tongji Medical College of Huazhong University of Science and Technology, Wuhan 430030, China; alimaalim1@hotmail.com (A.A.M.); wzhneuros@hust.edu.cn (Z.W.); 2 Sino-German Neuro-Oncology Molecular Laboratory, Tongji Hospital of Tongji Medical College of Huazhong University of Science and Technology, Wuhan 430030, China; 3 Hubei Key Laboratory of Neural Injury and Functional Reconstruction, Huazhong University of Science and Technology, Wuhan 430030, China

**Keywords:** meningioma, receptor for activated C kinase 1 (RACK1), casein kinase 2 (CK2), CSNK2B, cell cycle, harringtonolide (HA)

## Abstract

**Simple Summary:**

Malignant meningiomas have high aggressiveness and recurrence rates and a poor prognosis, with no clear pharmacological treatment available. The study aims to investigate the progression mechanism of malignant meningiomas and the targets of intervention. RACK1 and CSNK2B have been shown to promote tumor progression but their roles in meningiomas are not clear. In this study, we will investigate the roles of RACK1-CSNK2B in meningiomas and search for targets to inhibit the progression of meningiomas.

**Abstract:**

Higher-grade meningiomas (WHO grade II and III) are characterized by aggressive invasiveness and high postoperative recurrence rates. The prognosis remains inadequate even with adjuvant radiotherapy and currently there is no definitive pharmacological treatment strategy and target for malignant meningiomas. This study aims to unveil the mechanisms driving the malignant progression of meningiomas and to identify potential inhibitory targets, with significant clinical implications. Implementing techniques such as protein immunoprecipitation, mass spectrometry, RNA interference, and transcriptome sequencing, we investigated the malignancy mechanisms in meningioma cell lines IOMM-LEE and CH157-MN. Additionally, in vivo experiments were carried out on nude mice. We discovered a positive correlation between meningioma malignancy and the levels of the receptor for activated C kinase 1 (RACK1), which interacts with CSNK2B, the β subunit of casein kinase 2 (CK2), inhibiting its ubiquitination and subsequent degradation. This inhibition allows CK2 to activate the NF-κb pathway, which increases the transcription of CDK4 and cyclin D3, resulting in the transition of the cell cycle into the G2/M phase. The RACK1 inhibitor, harringtonolide (HA), significantly suppressed the malignant tendencies of meningioma cells. Our study suggests that RACK1 may play a role in the malignant progression of meningiomas, and therefore, targeting RACK1 could emerge as an effective strategy for reducing the malignancy of these tumors.

## 1. Introduction

Meningiomas are the most frequently reported primary CNS tumors, comprising ~36.1% of all CNS tumors, with an incidence of 7.61/100,000 [1], with a majority being benign and more prevalent among female patients [2,3]. Often asymptomatic, some meningiomas can lead to symptoms such as increased intracranial pressure and compression of the cranial nerves, leading to focal dysfunction and epilepsy. Surgical resection is the first choice of treatment, and the outcome is relatively good. However, a small proportion of meningiomas have a malignant tendency (WHO grade II and III) which is characterized by strong aggressiveness, a high rate of postoperative recurrence, and a poor prognosis even when combined with radiotherapy [4,5]. Currently, there are no definitive pharmacological treatments [6,7]. The mechanisms underlying the malignant progression of meningiomas remain unclear, although differences in gene expression have been noted between higher and less malignant meningiomas [8].

The cell cycle is a complex process divided into G1/G0, S, and G2/M phases, involving multiple genes [9]. The abnormal expression of genes associated with any phase can lead to cell cycle disorders, subsequently affecting cell proliferation and inducing various behavioral changes [10,11]. Several studies have shown that alterations in the cell cycle can lead to changes in the behavior of tumor cells [12,13] Furthermore, studies indicate that the overexpression of certain cell cycle-related genes may contribute to the malignant progression of meningiomas [14,15].

RACK1 is a kind of scaffolding protein that binds a variety of signaling molecules and anchors and stabilizes related proteins. It often binds to kinases and other proteins, affecting a variety of signaling pathways [16,17,18]. Some studies have shown that RACK1 is closely related to the progression of various tumors [19,20]. RACK1 could enhance the ability of self-renewal and chemoresistance of cancer stem cells in human hepatocellular carcinoma [19] and was reported to facilitate the development and lymph node metastasis of cervical cancer [21]. Besides, RACK1 may promote the ability of proliferation and the tendency of invasion and metastasis of breast cancer [22], and the decrease of ubiquitin degradation of RACK1 in ovarian cancer leads to the enhancement of the proliferation, migration, and invasion of ovarian cancer cells [23]. In the central nervous system, some studies have shown that RACK1 promotes the proliferation and invasion of glioma cells and may also affect the differentiation and apoptosis of glioma cells [24,25], but so far there are no studies on RACK1 in meningiomas. CSNK2B is a regulatory subunit of casein kinase 2, which regulates the catalytic activity of this kinase [26,27]. Some studies have shown that CSNK2B can activate the NF-κB pathway [28], which in turn promotes the expression of cell cycle-related proteins and facilitates the transformation of the cell cycle [23]. This process is thought to drive the proliferation of tumor cells and contribute to their malignant progression. However, the role of CSNK2B in the context of meningiomas has yet to be demonstrated.

## 2. Materials and Methods

### 2.1. Human Meningioma Samples

From June 2019 to November 2022, a total of 15 meningioma samples were acquired from patients who underwent surgery at the Department of Neurosurgery, Tongji Hospital of Tongji Medical College, Huazhong University of Science and Technology. The included participants comprised 9 females and 6 males, with an average age of 45.8 years (ranging from 21 to 67 years) at the time of surgery. The research protocol was approved by the ethical committees at Tongji Hospital, Tongji Medical College, Huazhong University of Science and Technology. Written informed consents were obtained from all patients involved in the study.

### 2.2. Cell Lines and Primary Cell Culture

The IOMM-LEE and CH157-MN cell lines, procured from the American Type Culture Collection and confirmed to be free of mycoplasma contamination using the Mycoplasma Stain Assay Kit (Beyotime, cat no. C0296, China), were cultured in DMEM medium (Gibco, Thermo Fisher, Waltham, MA, USA) supplemented with 10% fetal bovine serum, 1% streptomycin, and 1% penicillin. The cultures were maintained at 37 °C in a humidified atmosphere of 95% air and 5% CO_2_, with regular medium changes every 3 days.

### 2.3. Tumor Xenograft Experiments

For the in vivo experiments, we conducted xenograft models using IOMM-LEE cells. In brief, nude mice were randomly divided into multiple groups and received a subcutaneous injection of 1 × 10 ^7^ cells in their right axilla. The mice were intraperitoneally administered either PBS (CTRL) or HA (1 mg/kg) every 3 days after a IOMM-LEE cell injection. The tumor sizes were measured and documented every 3 days post-injection (volume = 0.5 × length × width^^2^). After a 15-day period post-injection, the tumors were excised. The excised tumor samples were then subjected to formalin fixation and paraffin embedding for subsequent analysis.

### 2.4. Cell Cycle Assay

Cell cycle progression and apoptosis were investigated using flow cytometry. For cell cycle analysis, a PI Cell Cycle detection kit (Yeasen, Shanghai, China) was used. The cells were digested with 0.25% trypsin, then centrifuged at a speed of 1000 rpm/min for 5 min, and the supernatant was discarded. After washing once with precooled PBS, the cells were mixed gently with 70% precooled ethanol and fixed overnight at 4 °C. Following another centrifugation at 1000 rpm for 5 min, the cells were resuspended in 1 mL of precooled PBS and centrifuged again under the same conditions. To the resuspended cells, 10 μL of propidium iodide storage solution and 10 μL of RNase A solution were added to 0.5 mL of staining buffer. The cell suspension was then mixed gently with 0.5 mL of the propidium iodide staining solution and incubated in the dark at 37 °C for 30 min. After filtration through a 400-mesh sieve, the cells were analyzed using a CytoFLEX V5-B5-R3 Flow Cytometer (13 Detectors, 3 Lasers) (Beckman, CA, USA). Data were analyzed with FlowJo v10 software (BD, Ashland, VA, USA).

### 2.5. Colony Formation Assay

The cells were initially plated in 6-well plates at a density of 1000 cells per well. After 24 h, the medium was substituted with a fresh complete medium. Then the cells were cultured for a period of 2 weeks, with a weekly medium renewal. After a 2-week incubation period, the colonies were fixed using 4% paraformaldehyde and stained with crystal violet.

### 2.6. Immunohistochemical Staining

Rehydrated tissue sections underwent blocking with 5% bovine serum albumin (BSA, ServiceBio, Wuhan, China) and subsequent staining with the primary antibody against RACK1 (27592-1-AP, Proteintech, Wuhan, China) at 4 °C for 12 h. Biotinylated anti-rabbit IgG (Boster, Wuhan, China) served as the secondary antibody for immunohistochemistry (IHC). Following this, streptavidin–biotin complex (SABC) (Boster, Wuhan, China) and DAB (DAB substrate with DAB chromagen at a ratio of 1 mL to 20 µL) were added for visualization, and hematoxylin was employed for nuclear staining. The microscopy used for observing all section staining results was from Olympus, Tokyo, Japan. ImageJ was utilized for analyzing the extent of the positive staining.

### 2.7. ELISA Assay

Blood samples of the patients were collected in heparin anticoagulated tubes prior to surgery and were subsequently stored at 4 °C. Then, the blood samples underwent centrifugation at 1000× *g* to obtain the serum within 24 h. The obtained serum was then stored at −20 °C. The concentrations of RACK1 and CSNK2B in the patients’ serum were determined using ELISA assays, following the manufacturer’s instructions. The RACK1 ELISA kit (SBJ-H1961, Sbj bio, Nanjing, China) and the CSNK2B ELISA kit (ZY-62657H, Zeye bio, Shanghai, China) were employed for the respective analyses. The standard curves of ELISA assay were fitted using Origin pro 2019b (Figure S1).

### 2.8. Luciferase Reporter Assay

IOMM-LEE cells were initially seeded into 96-well plates; after 24 h of incubation, the cell confluence reached 60–70%. Subsequently, the cells underwent co-transfection with NF-κB luciferase reporter gene plasmids (11501ES03, Yeasen, China) and Rinilla luciferase reporter gene plasmids (pGMLR-TK luciferase reporter gene plasmids) (11557ES03, Yeasen, China) using Lipofectamine 2000 (Invitrogen, Carlsbad, CA, USA) as per the manufacturer’s instructions. Following a 24-hour period, the Dual-luciferase Reporter Assay System (11402ES60, Yeasen, China) was employed to measure firefly and Renilla luciferase activities, and the results were recorded using Synerg 2 (Bio Tek, Shoreline, WA, USA). The Supplementary Material (Figure S2) provides details on the plasmids used.

### 2.9. Plasmid Construction and Transfection

The shRNAs plasmids and overexpression plasmids were constructed by Genechem (Shanghai, China). The plasmids were introduced into 200 μL Opti-MEM medium (Gibco, Thermo Fisher, Waltham, MA, USA). Transfection reagents (Lipo3000 4 μg and P3000 4 μg) (Invitrogen, Thermo Fisher, Waltham, MA, USA) were combined with 200 μL Opti-MEM medium. Following a 20-minute incubation period, the mixture was added to 293T cells. After 6–8 h, the medium was replaced with 2 mL DMEM medium (10% FBS). The virus solution was harvested 72 h later, filtered using a 0.22 μm filter, and applied to GH3 or GH4 cells. Another 6–8 h later, the medium was once again replaced with 2 mL of DMEM (10% FBS). To establish a stable transgenic cell line, selection with 2.5 mg/mL Puromycin was carried out for 3 days, commencing 72 h after infection.

### 2.10. Western Blot

The protein concentrations of the cells were determined using a BCA kit. Subsequently, the protein samples were diluted with 5× sample buffer solution and underwent electrophoresis on a 12% separation gel for 90 min. After electrophoresis, the gels were transferred to PVDF membranes (Immobilon^®^, Merck Millipore, Dublin, Ireland), which were then blocked with 1× PBS containing 5% non-fat dried milk for 1 h at room temperature. Incubation with primary antibodies (RACK1, CSNK2B, P65, p-P65, IKK, p-IKK, IκBα, phospho-IκBα) at specified dilutions (all at 1:1000, except GAPDH at 1:5000, with suppliers as indicated) occurred at 4 °C overnight. After washing, the membranes were exposed to HRP-conjugated anti-rabbit or mouse secondary antibody (1:5000, Proteintech, Wuhan, China) for 2 h at room temperature. Subsequently, the membranes were developed and imaged using a Gene Gnome exposure instrument. The protein expression levels were quantified through densitometry and normalized to GAPDH levels.

### 2.11. Quantitative Real-Time PCR

The total RNA of the cells was extracted using TRIzol (Servicebio, Wuhan, China). The extracted RNA was then reverse transcribed into cDNA, utilizing the Hifair® III 1st Strand cDNA Synthesis Kit (Yeasen, Shanghai, China), following the manufacturer’s datasheet. Subsequently, the gene products were amplified through quantitative real-time PCR on an ABI-Prism 7500 Real-Time PCR System (Applied Biosystems, Carlsbad, CA, USA) using Hieff® qPCR SYBR Green Master Mix (Low Rox Plus) (Yeasen, Shanghai, China). The mRNA expression levels were normalized to the internal standard GAPDH. Data analysis was conducted using the 2^(−ΔΔCt) method.

### 2.12. Statistical Analyses

The data are presented as the mean ± standard deviation. Statistical analysis and chart creation were performed using GraphPad Prism 9.0, while picture clipping was accomplished using Adobe Photoshop CC2018. Multiple comparisons were assessed through one-way ANOVA followed by Tukey’s test, and comparisons between the two groups were analyzed using a non-paired t-test. A significance level of *p* < 0.05 was considered statistically significant.

## 3. Results

### 3.1. RACK1 Expression Level Positively Correlates with the Malignancy of Meningiomas

**Figure 1:**
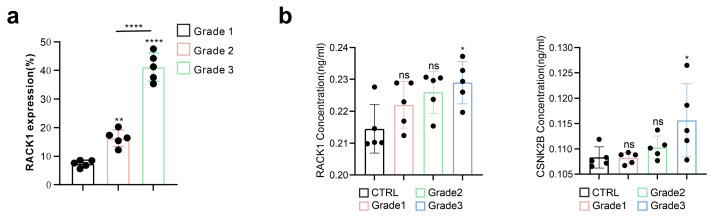
**The** RACK1 expression level positively correlates with the malignancy of meningiomas. Immunohistochemical staining of meningioma sections (* *p* < 0.05, ** *p* < 0.01, **** *p* < 0.0001, ns: not statistically significant).

To investigate the correlation between RACK1 expression and the malignancy of meningiomas, we collected tumor tissues from meningiomas of various WHO grades for immunohistochemical and HE staining (Figure S3). Our findings indicate that RACK1 expression levels were higher in meningiomas with a greater degree of malignancy, and that there were significant differences in RACK1 levels among the different WHO grades (Figure 1a). Besides, we found that the levels of RACK1 and CSN2KB in the serum samples of patients with malignant meningiomas (especially WHO grade3) were higher than patients without meningioma (Figure 1b).

### 3.2. RACK1 Promotes the Proliferation and Migration Ability of Meningioma Cells and Affects the Cell Cycle

**Figure 2:**
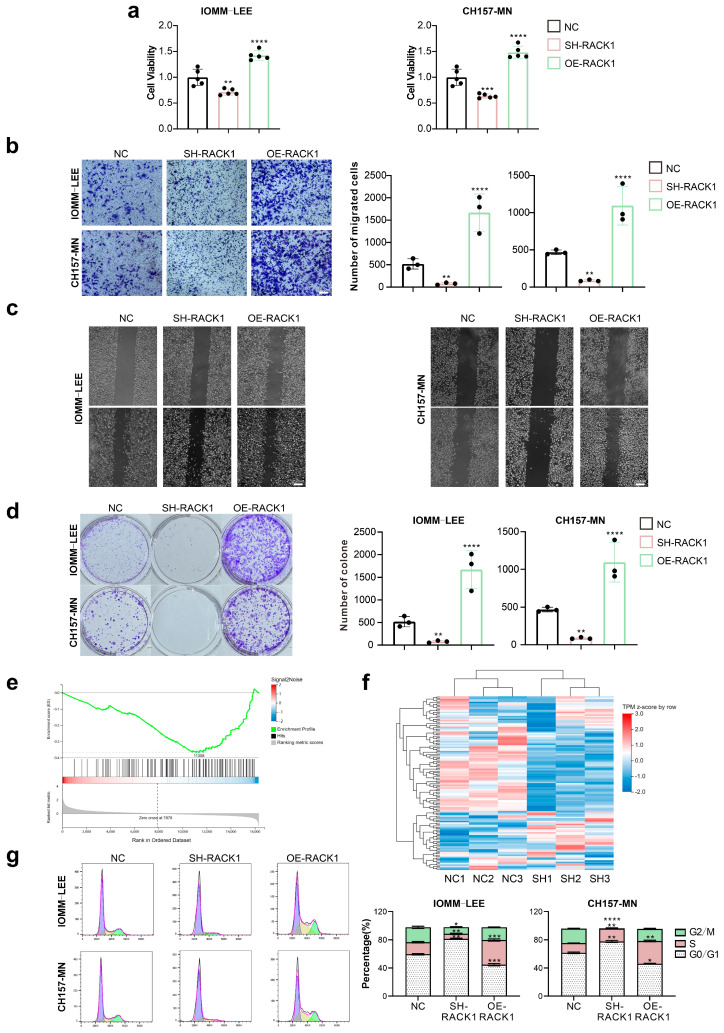
RACK1 promotes the proliferation and migration ability of meningioma cells and affects the cell cycle. (**a**) Cell viability of IOMM-LEE and CH157-MN is detected by CCK-8 assay (** *p* < 0.01, *** *p* < 0.001, **** *p* < 0.0001); (**b**) Transwell assay of IOMM-LEE and CH157-MN (** *p* < 0.01, **** *p* < 0.0001); (**c**) Scratch assay of IOMM-LEE and CH157-MN; (**d**) Colony assay of IOMM-LEE and CH157-MN (** *p* < 0.01, **** *p* < 0.0001); (**e**,**f**) GSEA pathway enrichment analysis of cell cycle pathway; (**g**) Cell cycle of IOMM-LEE and CH157-MN is detected by flow cytometry (* *p* < 0.05, ** *p* < 0.01, *** *p* < 0.001, **** *p* < 0.0001).

As we discovered, RACK1 expression is elevated in more malignant meningiomas. Consequently, we aimed to investigate the role of RACK1 in the malignant progression of meningiomas and to determine its effects on this process. We altered RACK1 expression in the IOMM-LEE and CH157-MN cells, which are widely utilized human malignant meningioma cell lines, through knockdown and overexpression, respectively. We then observed the effects on cell behavior. CCK-8 assays revealed that RACK1 knockdown significantly reduced cell viability, whereas cells overexpressing RACK1 showed a marked increase in viability (Figure 2a). Furthermore, cell proliferation and migration were significantly decreased following RACK1 knockdown, while cells with RACK1 overexpression exhibited a significant increase in these abilities, as evidenced by colony formation, transwell, and scratch assays (Figure 2b–d). 

To explore how RACK1 affects the malignant progression of meningioma cells, we sequenced the transcriptome of IOMM-LEE cells and IOMM-LEE cells with RACK1 knockdown. We then performed GSEA pathway enrichment analysis on the transcriptome sequencing results, and we found that the cell cycle pathway was significantly downregulated in cells with RACK1 knockdown (Figure 2e–f). Subsequently, we examined the effect of RACK1 on the cell cycle of meningioma cells using flow cytometry. We observed that after RACK1 knockdown, the proportion of cells in the S phase and G2/M phase was significantly decreased, and the proportion of cells in the G0/G1 phase was significantly increased. In contrast, the overexpression of RACK1 led to a significant increase in the proportion of cells in the S phase and G2/M phase, and a decrease in the proportion of cells in the G0/G1 phase (Figure 2g).

### 3.3. RACK1 Inhibits its Ubiquitination Degradation by Binding to CSNK2B

**Figure 3:**
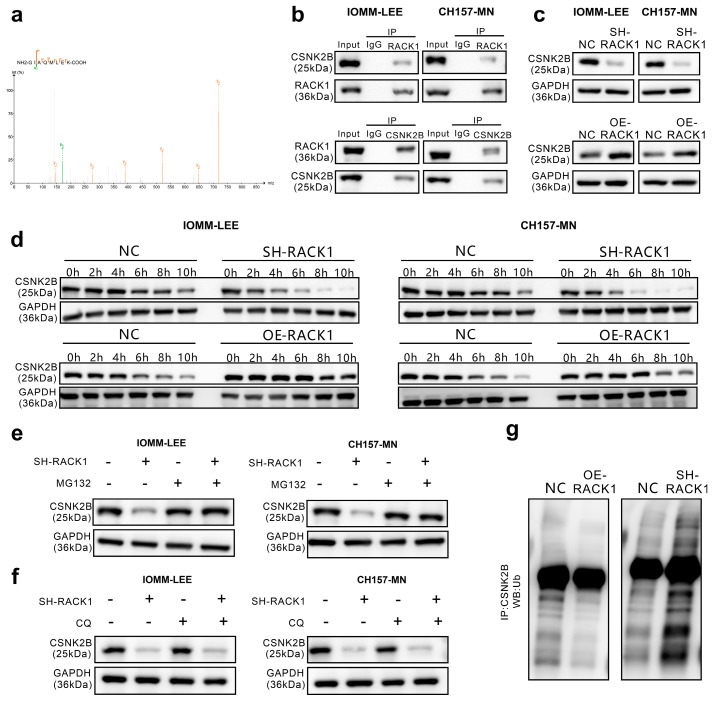
RACK1 inhibits its ubiquitination degradation by binding to CSNK2B. (**a**) Whole-cell extracts obtained from IOMM-LEE cells were incubated with an anti-RACK1 antibody. The purified protein was subjected to analysis using LC-MS/MS; (**b**) Western blot was conducted on the whole-cell lysates (input), Co-IP complexes captured using anti-CSNK2B antibody and anti-CSNK2B antibody in both IOMM-LEE and CH157-MN cells; (**c**) Western blot of IOMM-LEE and CH157-MN lysates whose RACK1 were knocked down or overexpressed; (**d**) Western blot of IOMM-LEE and CH157-MN lysates which were used with cycloheximide (CHX) to inhibit protein synthesis; (**e**) Western blot of IOMM-LEE and CH157-MN lysates which were used with MG132; (**f**) Western blot of IOMM-LEE and CH157-MN lysates which were used with CQ; (**g**) Western blot of Co-IP complex captured with anti-CSNK2B antibody in IOMM-LEE cells to detect the ubiquitin levels.

The receptor for activated C kinase 1 (RACK1) is a multifaceted signaling adaptor that participates in multiple biological events [16]. To investigate how RACK1 affects the proliferation, migration, and cell cycle functions of meningioma cells, a Co-IP assay coupled with LC-MS/MS was employed to identify proteins interacting with RACK1. Among all the interacting proteins, CSNK2B has the highest LFQ intensity log2 FC (LFQ intensity RACK1/LFQ intensity IgG) except the Bait (RACK1). The list of interacted proteins is in the Supplementary Material (Table S1). CSNK2B is the regulatory subunit of casein kinase II and is known to regulate kinase activity through the phosphorylation of serine/threonine residues [26] (Figure 3a).

First, to validate the results of the LC-MS/MS assay, we confirmed the binding relationship between RACK1 and CSNK2B through immunoprecipitation (Figure 3b). Furthermore, we verified by Western blot that there is a significant correlation between the expression levels of CSNK2B and RACK1 (Figure 3c). Then, using cycloheximide (CHX) to inhibit protein synthesis, we observed by Western blot that CSNK2B degradation was significantly accelerated in meningioma cells with RACK1 knockdown, whereas degradation was significantly slowed in those cells overexpressing RACK1 (Figure 3d). Additionally, the protease inhibitor MG132 was found to counteract the downregulation of CSNK2B induced by RACK1 knockdown (Figure 3e), while chloroquine did not have this effect (Figure 3f). We also discovered that the overexpression of RACK1 attenuated the ubiquitination of CSNK2B by immunoprecipitation, whereas RACK1 knockdown increased CSNK2B ubiquitination (Figure 3g). Therefore, we hypothesized that RACK1 may inhibit the ubiquitin-mediated degradation of CSNK2B by binding to CSNK2B.

### 3.4. CSNK2B Promotes the Proliferation and Migration Ability of Meningioma Cells and Affects the Cell Cycle

**Figure 4:**
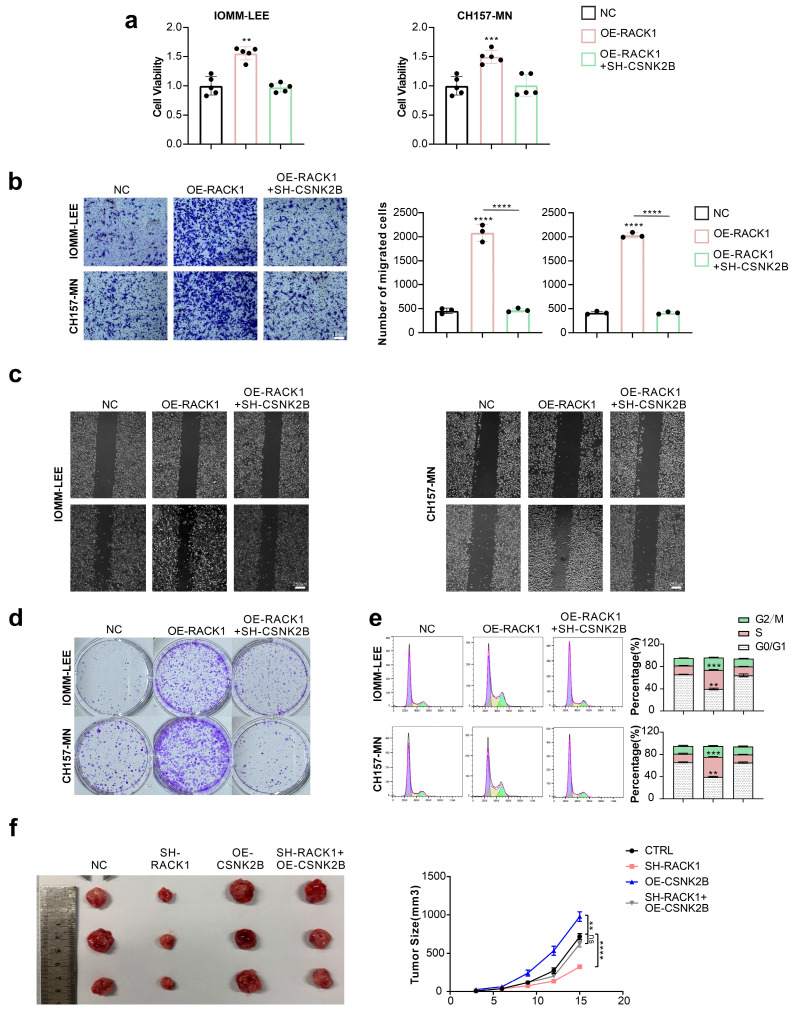
CSNK2B promotes the proliferation and migration ability of meningioma cells and affects the cell cycle. (**a**) Cell viability of IOMM-LEE and CH157-MN detected by CCK-8 assay (** *p* < 0.01, *** *p* < 0.001); (**b**) Transwell assay of IOMM-LEE and CH157-MN (*** *p* < 0.001, **** *p* < 0.0001); (**c**) Scratch assay of IOMM-LEE and CH157-MN cells; (**d**) Colony formation assay of IOMM-LEE and CH157-MN cells; (**e**) Flow cytometry of IOMM-LEE and CH157-MN cells was conducted to detected the cell cycle. (** *p* < 0.01, *** *p* < 0.001); (**f**) Nude mice received a subcutaneous injection of 1 × 10^^7^ IOMM-LEE cells in their right axilla, and the tumor sizes were measured every 3 days (volume = 0.5 × length × width^^2^) (N = 3) (** *p* < 0.01, **** *p* < 0.0001).

To investigate the effect of CSNK2B on the behavior of meningioma cells, we altered the expression of RACK1 and CSNK2B in IOMM-LEE and CH157-MN cells. We assessed the changes in cell viability as well as proliferation and migration ability by CCK-8 assay, colony formation assay, Transwell assay, and scratch assay (Figure 4a–d). The knockdown of CSNK2B counteracted the enhanced effects of RACK1 overexpression on cell viability, proliferation, and migration. Furthermore, flow cytometry revealed that CSNK2B knockdown attenuated the effects of RACK1 overexpression on the meningioma cell cycle (Figure 4e). Additionally, the subcutaneous implantation of tumors in nude mice showed that, compared to the control group, meningioma cells with RACK1 knockdown formed smaller tumors, while those overexpressing CSNK2B developed larger tumors. However, cells with both RACK1-KO and CSNK2B-OE formed tumors with sizes not significantly different from the control group (Figure 4f).

### 3.5. CSNK2B Promotes the Expression of CDK4 and Cyclin D3 by Activating the NF-κB Pathway

**Figure 5:**
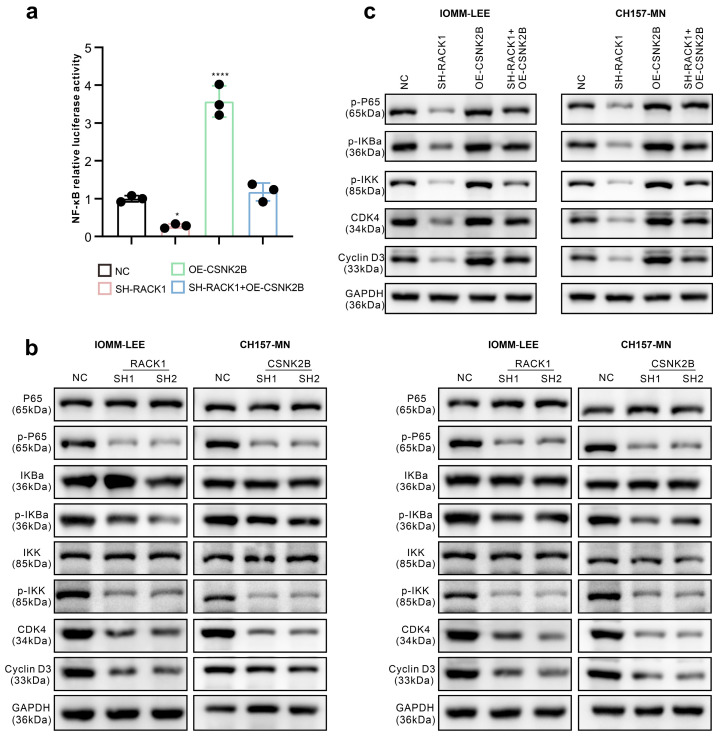
CSNK2B activates the NF-κB pathway. (**a**) NF-κB transcriptional activity of IOMM-LEE is detected by dual luciferase assay (* *p* < 0.05, **** *p* < 0.0001); (**b**,**c**) Western blot of IOMM-LEE and CH157-MN lysates to detect the expression of NF-κB pathway protein.

First, we performed KEGG pathway enrichment of the proteins interacting with RACK1 and found that the NF-κB pathway had the smallest p-value (*p* = 0.0025), so we inferred that the NF-κB pathway may be closely linked to RACK1-CSNK2B. The results of KEGG enrichment are displayed in the Supplementary Material (Table S2, Figure S4). Besides, in previous studies, CSNK2B has been shown to affect tumor progression by activating the NF-κB pathway [28,29]. Therefore, we evaluated the effect of RACK1 and CSNK2B expression levels on NF-κB transcriptional activity using a dual luciferase assay. The results showed that the knockdown of RACK1 significantly decreased the NF-κB transcriptional activity, whereas the overexpression of CSNK2B resulted in a significant increase. However, the simultaneous knockdown of RACK1 and the overexpression of CSNK2B resulted in no significant change in NF-κB transcriptional activity compared to the control group (Figure 5a). Transcriptome sequencing data analysis revealed that RACK1 knockdown significantly downregulated the cell cycle pathway in meningioma cells, with CDK4 and cyclin D3 being the most affected. Therefore, we assessed the effects of RACK1 and CSNK2B on the NF-κB pathway and CDK4 and cyclin D3 expression levels by western blot. We observed that the knockdown of both RACK1 and CSNK2B downregulated the protein levels of p-p65, p-IκBα, p-IKK, CDK4, and cyclin D3 (Figure 5b). On the other hand, the overexpression of CSNK2B, following the knockdown of RACK1, counteracted the downregulation of p-p65, p-IκBα, p-IKK, CDK4, and cyclin D3 caused by the knockdown of RACK1 (Figure 5c).

**Figure 6:**
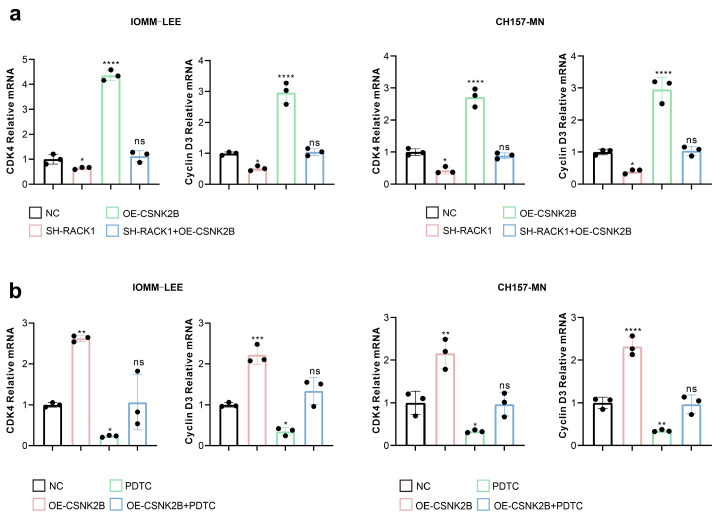
CSNK2B promotes the expression of CDK4 and cyclin D3. (**a**,**b**) mRNA levels of CDK4 and cyclin D3 of IOMM-LEE and CH157-MN cells are detected by qPCR (* *p* < 0.05, ** *p* < 0.01, *** *p* < 0.001, **** *p* < 0.0001).

Furthermore, we explored the effects of RACK1 and CSNK2B on CDK4 and cyclin D3 mRNA levels using qPCR. Our findings showed that the knockdown of RACK1 down-regulated CDK4 and cyclin D3 mRNA levels. However, the overexpression of CSNK2B, following RACK1 knockdown, reversed the downregulation of CDK4 and cyclin D3 mRNA levels (Figure 6a). Additionally, we discovered that the NF-κB inhibitor PDTC counteracted the stimulatory effect of CSNK2B overexpression on CDK4 and cyclin D3 mRNA expression (Figure 6b).

### 3.6. RACK1 Inhibitor HA Suppresses Value-Added Migration of Meningioma Cells

**Figure 7:**
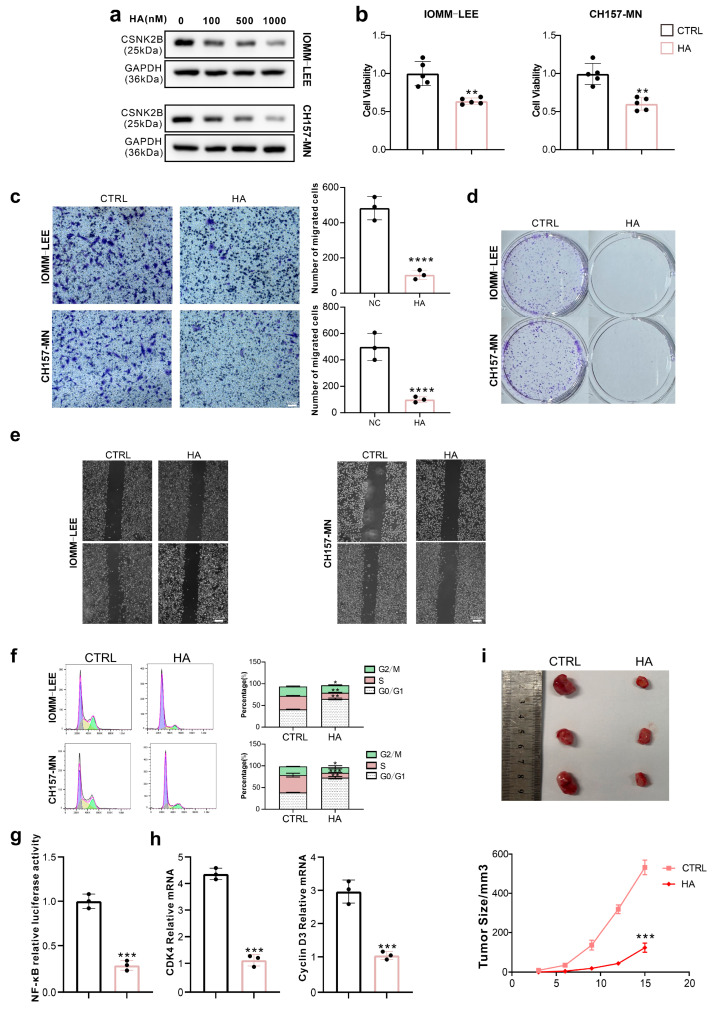
RACK1 inhibitor HA suppresses the value-added migration of meningioma cells. (**a**) Western blot of IOMM-LEE and CH157-MN lysates to detect the effect of HA on the expression of CSNK2B; (**b**) Cell viability of IOMM-LEE and CH157-MN is detected by CCK-8 assay (** *p* < 0.01); (**c**) Transwell assay of IOMM-LEE and CH157-MN (**** *p* < 0.0001); (**d**) Colony formation assay of IOMM-LEE and CH157-MN; (**e**) Scratch assay of IOMM-LEE and CH157-MN; (**f**) Cell cycle of IOMM-LEE and CH157-MN is detected by flow cytometry (* *p* < 0.05, ** *p* < 0.01, *** *p* < 0.001); (**g**) NF-κB transcriptional activity of IOMM-LEE detected by dual luciferase assay (*** *p* < 0.001); (**h**) mRNA levels of CDK4 and cyclin D3 of IOMM-LEE and CH157-MN cells are detected by qPCR (*** *p* < 0.001); (**i**) IOMM-LEE cells were used to form tumors under the axils of nude mice. The tumor size was measured every 3 days (volume = 0.5 × length × width^^2^) (N = 3) (*** *p* < 0.001).

Harringtonolide (HA) is a RACK1 inhibitor [30], and while some studies have demonstrated its potential anti-tumor effects [31,32], its effects on meningiomas have not been studied. Initially, we found that HA inhibits CSNK2B protein expression in meningioma cells in a dose-dependent manner, as shown by Western blot analysis (Figure 7a). Subsequent assays, including CCK-8, colony formation, Transwell, and scratch assays, revealed that HA significantly inhibits cell viability, proliferation, and migration in meningioma cells (Figure 7b–e). Flow cytometry analysis showed that HA significantly decreased the proportion of cells in the S and G2/M phases while increasing the proportion in the G0/G1 phase (Figure 7f). Moreover, HA was found to significantly inhibit NF-κB transcriptional activity using a dual luciferase assay (Figure 7g) and to reduce the transcriptional levels of CDK4 and cyclin D3 by qPCR (Figure 7h). Furthermore, HA significantly inhibits tumor growth in subcutaneous implantations in nude mice (Figure 7i).

## 4. Discussion

Meningiomas with malignant tendency (WHO grade II and III) have a high rate of postoperative recurrence and poor prognosis even when combined with radiation therapy [33]; there is no clear chemotherapeutic regimen for malignant meningiomas, and there is no clear mechanism to explain the malignant progression of meningiomas [6,7]. Therefore, it is of great clinical significance to explore the mechanisms of the malignant progression of meningiomas and to find potential therapeutic targets.

It has been shown that RACK1 is closely related to tumor progression [19,21,22,23,24,25] due to its modulation of multiple signaling pathways including PKC, receptor tyrosine kinase/PI3K/AKT, ERK/MAPK, STAT3, Src/FAK, NF-κB, Wnt/β-catenin, and RhoA/Rho pathway [26,34,35,36,37]. RACK1 affects various behaviors such as cell cycle, cell proliferation, migration and invasion, epithelial mesenchymal transition, and tumor cell differentiation via these signal pathways. Many of these mechanisms are also involved in meningioma development and progression, such as the cell cycle [14,38], PI3K-AKT pathway [39], and STAT3 pathway [40]. However, the role of RACK1 in meningiomas has not yet been explored. In this study, we collected meningioma specimens of different WHO grades and performed immunohistochemical staining. We found a significant correlation between the malignancy of meningiomas and the expression level of RACK1, a scaffolding protein that anchors and maintains structural stability for other proteins and is known to interact with various kinases to regulate multiple signaling pathways. 

CSNK2B, as a regulatory subunit of casein kinase 2, regulates the enzyme’s catalytic activity. It has been shown to play a critical role in activating the NF-κB pathway [28], which influences tumor progression [41,42]. Our study found that in malignant meningioma cells, RACK1 interacts with CSNK2B and inhibits its ubiquitination and degradation by binding to it. This interaction promotes the expression of cell cycle-related genes, including CDK4 and cyclin D3, via the activation of the NF-κB pathway. CDK4 and cyclin D3 drive meningioma cells into the S phase and enhance cell proliferation and migration [43,44]. 

In our study, inhibiting RACK1 and CSNK2B expression significantly suppressed the proliferation and migration ability of meningioma cells. Furthermore, HA, an inhibitor of RACK1 [30], significantly inhibited the promotion of RACK1 on the malignancy of meningiomas.

In this study, we found that the RACK1-CSNK2B complex regulates the NK-KB pathway and affects the malignant progression of meningiomas by promoting cell cycle transition. However, the regulation of signaling pathways in cells is complex, and, besides this, there may be other signaling pathways regulated by RACK1-CSNK2B that are involved in the malignant progression of meningiomas and that require further exploration.

## 5. Conclusions

Our study revealed that RACK1 prevents the ubiquitination and subsequent degradation of CSNK2B by binding to it. This interaction promotes the transcription of cell cycle genes CDK4 and cyclin D3 through the activation of the NF-κB pathway, thereby enhancing the proliferation of meningioma cells. Moreover, we found that the inhibition of RACK1 using Harringtonolide (HA) inhibits the progression of malignant meningiomas.

## Data Availability

The data presented in this study are available on request from the corresponding authors.

## Supplementary Materials

The following supporting information can be downloaded at: https://www.mdpi.com/article/10.3390/cancers16040767/s1, Figure S1: The standard curves of ELISA assay; Figure S2: NF-κB luciferase reporter gene plasmid and Rinilla luciferase reporter gene plasmid (pGMLR-TK luciferase reporter gene plasmid); Figure S3: Immunohistochemical and HE staining of different WHO grades of meningiomas; Table S1: The list of RACK1-interaction proteins identified by LC-MS/MS assay; Table S2: List of KEGG pathway of proteins interacting with RACK1; Figure S4: KEGG pathway enrichment of proteins interacting with RACK1; Figure S5: Original Western Blot images.
