# Supplementary material for: RACK1 Promotes Meningioma Progression by Activation of NF-κB Pathway via Preventing CSNK2B from Ubiquitination Degradation
Source: Cancers (Basel). 2024 Feb 13;16(4):767. doi: 10.3390/cancers16040767 (PMC10886518; doi:10.3390/cancers16040767)
Supplement: Supplementary file 1 [file cancers-16-00767-s001.zip › cancers-2847254-supplementary.pdf]

Supplementary Material

Figure S1

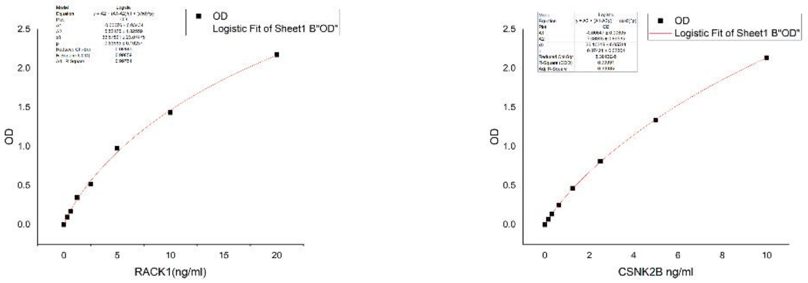

Figure S1. The standard curves of ELISA assay.

Figure S2

a

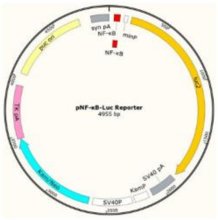

2) 质粒元件信息

NF-κB response element (NF-κB)	44-95
Minimal promoter (minP)	130-160
Luciferase reporter gene (luc2)	193-1845
SV40 late poly(A) signal (SV40 pA)	1880-2101
KamR early promoter (KamP)	2136-2260
SV40 enhancer and early promoter (SV40P)	2280-2618
Synthetic aminoglycoside phosphotransferase (Kam/Neo) coding region	2653-3471
herpesvirus thymidine kinase polyadenylation signal (Tk pA)	3472-3927
high-copy-number pUC origin of replication (puc ori)	4032-4699
Synthetic poly(A) signal (syn pA)	4802-4955

3) NF-κB response element 序列信息

1-50: TGAGCTCGCTAGCCTCGAGGATATGCGGGAATTTCCGGGGACTTTCCGGG

51-100: AATTTCCGGGGACTTTCCGGGAATTTCAAGATCTGGCCTCGGCGGCCAAG

pNF-κB-Luc 质粒测序引物

5' -CTAGCAAAATAGGCTGTCCC-3'

b

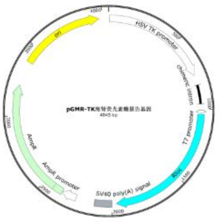

2) 质粒元件信息

NF-κB response element (NF-κB)	44-95
Minimal promoter (minP)	130-160
Luciferase reporter gene (luc2)	193-1845
SV40 late poly(A) signal (SV40 pA)	1880-2101
KamR early promoter (KamP)	2136-2260
SV40 enhancer and early promoter (SV40P)	2280-2618
Synthetic aminoglycoside phosphotransferase (Kam/Neo) coding region	2653-3471
herpesvirus thymidine kinase polyadenylation signal (Tk pA)	3472-3927
high-copy-number pUC origin of replication (puc ori)	4032-4699
Synthetic poly(A) signal (syn pA)	4802-4955

3) NF-κB response element 序列信息

1-50: TGAGCTCGCTAGCCTCGAGGATATGCGGGAATTTCCGGGGACTTTCCGGG

51-100: AATTTCCGGGGACTTTCCGGGAATTTCAAGATCTGGCCTCGGCGGCCAAG

pNF-κB-Luc 质粒测序引物

5' -CTAGCAAAATAGGCTGTCCC-3'

Figure S2. (a) NF-κB luciferase reporter gene plasmid; (b) Rinilla luciferase reporter gene plasmid (pGMLR-TK luciferase reporter gene plasmid).

Figure S3

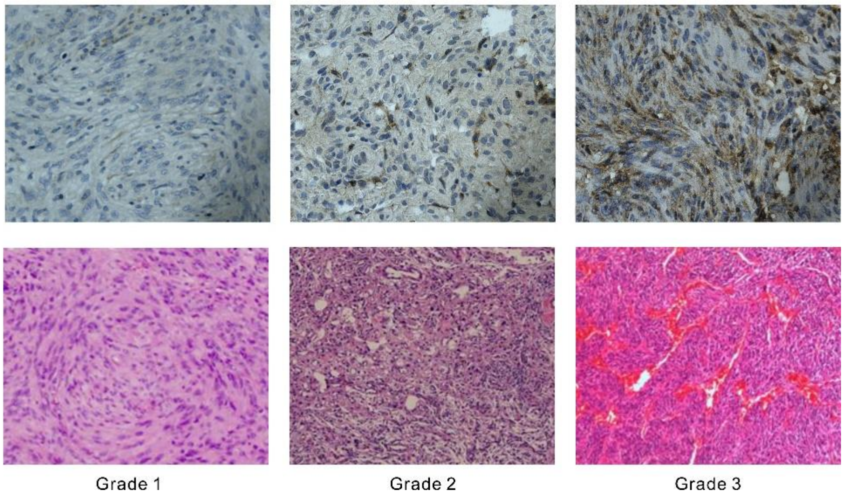

Figure S3. Immunohistochemical staining and HE staining of different WHO grades of meningiomas.

Table S1. The list of RACK1-interaction proteins identified by LC-MS/MS assay

Accession	Protein	Gene	Description	Mw(kDa)	Length	log2 intensity Rack1	log2 intensity IgG	log2 FC	Fold Change	Type
P63244	RACK1_HUMAN	RACK1	Receptor of activated protein C kinase 1 OS=Homo sapiens OX=9606 GN=RACK1 PE=1 SV=3	35.077	317	33.53322331	26.00859788	7.5246254	184.1356836	Bait
P67870	CSK2B_HUMAN	CSNK2B	Casein kinase II subunit beta OS=Homo sapiens OX=9606 GN=CSNK2B PE=1 SV=1	24.942	215	25.39381725	20.96659332	4.4272239	21.51429899	Interactor
Q9Y277	VDAC3_HUMAN	VDAC3	Voltage-dependent anion-selective channel protein 3 OS=Homo sapiens OX=9606 GN=VDAC3 PE=1 SV=1	30.659	283	24.01801268	19.64482034	4.3731923	20.72345064	Interactor
Q9Y305	ACOT9_HUMAN	ACOT9	Acyl-coenzyme A thioesterase 9, mitochondrial OS=Homo sapiens OX=9606 GN=ACOT9 PE=1 SV=2	49.902	439	24.25291947	19.94117577	4.3117437	19.85931142	Interactor
P11387	TOP1_HUMAN	TOP1	DNA topoisomerase 1 OS=Homo sapiens OX=9606 GN=TOP1 PE=1 SV=2	90.726	765	24.25688302	20.04985588	4.2070271	18.46891413	Interactor
Q14204	DYHC1_HUMAN	DYNC1H1	Cytoplasmic dynein 1 heavy chain 1 OS=Homo sapiens OX=9606 GN=DYNC1H1 PE=1 SV=5	532.408	4646	24.23461865	20.37589754	3.8587211	14.50744057	Interactor
O43310	CTIF_HUMAN	CTIF	CBP80/20-dependent translation initiation factor OS=Homo sapiens OX=9606 GN=CTIF PE=1 SV=1	67.587	598	22.9388179	19.18462222	3.7541957	13.49352781	Interactor
P49458	SRP09_HUMAN	SRP9	Signal recognition particle 9 kDa protein OS=Homo sapiens OX=9606 GN=SRP9 PE=1 SV=2	10.112	86	23.74088011	20.10584933	3.6350308	12.423767	Interactor

Q96AG4	LRC59_HUMAN	LRRC59	Leucine-rich repeat-containing protein 59 OS=Homo sapiens OX=9606 GN=LRRC59 PE=1 SV=1	34.93	307	23.87492733	20.24551033	3.629417	12.37551792	Interactor
Q96GV9	MACIR_HUMAN	MACIR	Macrophage immunometabolism regulator OS=Homo sapiens OX=9606 GN=MACIR PE=1 SV=1	23.083	206	23.36158616	19.76098568	3.6006005	12.13078056	Interactor
P00749	UROK_HUMAN	PLAU	Urokinase-type plasminogen activator OS=Homo sapiens OX=9606 GN=PLAU PE=1 SV=3	48.523	431	23.73912958	20.32191862	3.417211	10.68274837	Interactor
P10321	HLAC_HUMAN	HLA-C	HLA class I histocompatibility antigen, C alpha chain OS=Homo sapiens OX=9606 GN=HLA-C PE=1 SV=3	40.649	366	23.50191006	20.11943659	3.3824735	10.42859914	Interactor
P17096	HMGA1_HUMAN	HMGA1	High mobility group protein HMG-I/HMG-Y OS=Homo sapiens OX=9606 GN=HMGA1 PE=1 SV=3	11.676	107	23.24235946	20.19250781	3.0498516	8.281267782	Interactor
P24666	PPAC_HUMAN	ACP1	Low molecular weight phosphotyrosine protein phosphatase OS=Homo sapiens OX=9606 GN=ACP1 PE=1 SV=3	18.042	158	24.03246214	21.30719274	2.7252694	6.612837289	Interactor
O43791	SPOP_HUMAN	SPOP	Speckle-type POZ protein OS=Homo sapiens OX=9606 GN=SPOP PE=1 SV=1	42.132	374	25.102415	22.40036039	2.7020546	6.507279887	Interactor
Q9UDW1	QCR9_HUMAN	UQCR10	Cytochrome b-c1 complex subunit 9 OS=Homo sapiens	7.308	63	22.93569238	20.2354152	2.7002772	6.499267772	Interactor

			OX=9606 GN=UQCR10 PE=1 SV=3							
P43490	NAMPT_HUMAN	NAMPT	Nicotinamide phosphoribosyltransferase OS=Homo sapiens OX=9606 GN=NAMPT PE=1 SV=1	55.521	491	24.92406511	22.25390056	2.6701645	6.365017795	Interactor
Q16576	RBBP7_HUMAN	RBBP7	Histone-binding protein RBBP7 OS=Homo sapiens OX=9606 GN=RBBP7 PE=1 SV=1	47.82	425	22.69858768	20.15338685	2.5452008	5.836893847	Interactor
Q9BX40	LS14B_HUMAN	LSM14B	Protein LSM14 homolog B OS=Homo sapiens OX=9606 GN=LSM14B PE=1 SV=1	42.071	385	22.57715496	20.08995302	2.4872019	5.606894559	Interactor
P23743	DGKA_HUMAN	DGKA	Diacylglycerol kinase alpha OS=Homo sapiens OX=9606 GN=DGKA PE=1 SV=3	82.63	735	23.17366799	20.73240751	2.4412605	5.431160439	Interactor
Q71RC2	LARP4_HUMAN	LARP4	La-related protein 4 OS=Homo sapiens OX=9606 GN=LARP4 PE=1 SV=3	80.596	724	23.42649614	20.98615757	2.4403386	5.427690925	Interactor
Q9BSJ8	ESYT1_HUMAN	ESYT1	Extended synaptotagmin-1 OS=Homo sapiens OX=9606 GN=ESYT1 PE=1 SV=1	122.856	1104	22.43165675	20.03362813	2.3980286	5.270824351	Interactor
A2AJT9	BCLA3_HUMAN	BCLAF3	BCLAF1 and THRAP3 family member 3 OS=Homo sapiens OX=9606 GN=BCLAF3 PE=1 SV=1	83.871	711	22.31152217	19.91584352	2.3956786	5.262245822	Interactor
Q13610	PWP1_HUMAN	PWP1	Periodic tryptophan protein 1 homolog OS=Homo sapiens OX=9606	55.828	501	24.45274497	22.24244459	2.2103004	4.627716165	Interactor

			GN=PWP1 PE=1 SV=1							
P11177	ODPB_HUMAN	PDHB	Pyruvate dehydrogenase E1 component subunit beta, mitochondrial OS=Homo sapiens OX=9606 GN=PDHB PE=1 SV=3	39.233	359	22.52202687	20.38456278	2.1374641	4.399879765	Interactor
P29728	OAS2_HUMAN	OAS2	2'-5'-oligoadenylate synthase 2 OS=Homo sapiens OX=9606 GN=OAS2 PE=1 SV=3	82.431	719	22.67383354	20.62018796	2.0536456	4.151537062	Interactor

Table S2. List of KEGG pathway of proteins interacting with RACK1.

Term	Description	Class	Qe Ratio	Bg Ratio	P_value	Count	Rich Factor	Fold Enrichment	Accession	Gene
hsa04064	NF-kappa B signaling pathway	Environmental Information Processing	2|13	3|431	0.002482059	2	0.66666667	22.1025641	P67870;P00749	CSNK2B; PLAU
hsa04621	NOD-like receptor signaling pathway	Organismal Systems	3|13	11|431	0.003090536	3	0.27272727	9.04195804	Q9Y277;P29728;P43490	NAMPT; VDAC3; OAS2
hsa05162	Measles	Human Diseases	3|13	12|431	0.004048136	3	0.25	8.28846154	P29728;P67870;P63244	CSNK2B; OAS2;RACK1
hsa05208	Chemical carcinogenesis - reactive oxygen species	Human Diseases	3|13	16|431	0.009596315	3	0.1875	6.21634615	P24666;Q9Y277;Q9UDW1	UQCR10; ACP1;VDAC3
hsa05415	Diabetic cardiomyopathy	Human Diseases	3|13	17|431	0.011446834	3	0.17647059	5.85067873	Q9UDW1;Q9Y277;P11177	UQCR10; PDHB;VDAC3
hsa00564	Glycerophospholipid metabolism	Metabolism	1|13	1|431	0.030162413	1	1	33.1538462	P23743	DGKA
hsa04072	Phospholipase D signaling pathway	Environmental Information Processing	1|13	1|431	0.030162413	1	1	33.1538462	P23743	DGKA
hsa00740	Riboflavin metabolism	Metabolism	1|13	1|431	0.030162413	1	1	33.1538462	P24666	ACP1
hsa04610	Complement and coagulation cascades	Organismal Systems	1|13	1|431	0.030162413	1	1	33.1538462	P00749	PLAU
hsa04520	Adherens junction	Cellular Processes	2|13	11|431	0.039653415	2	0.18181818	6.02797203	P24666;P67870	CSNK2B; ACP1

Figure S4

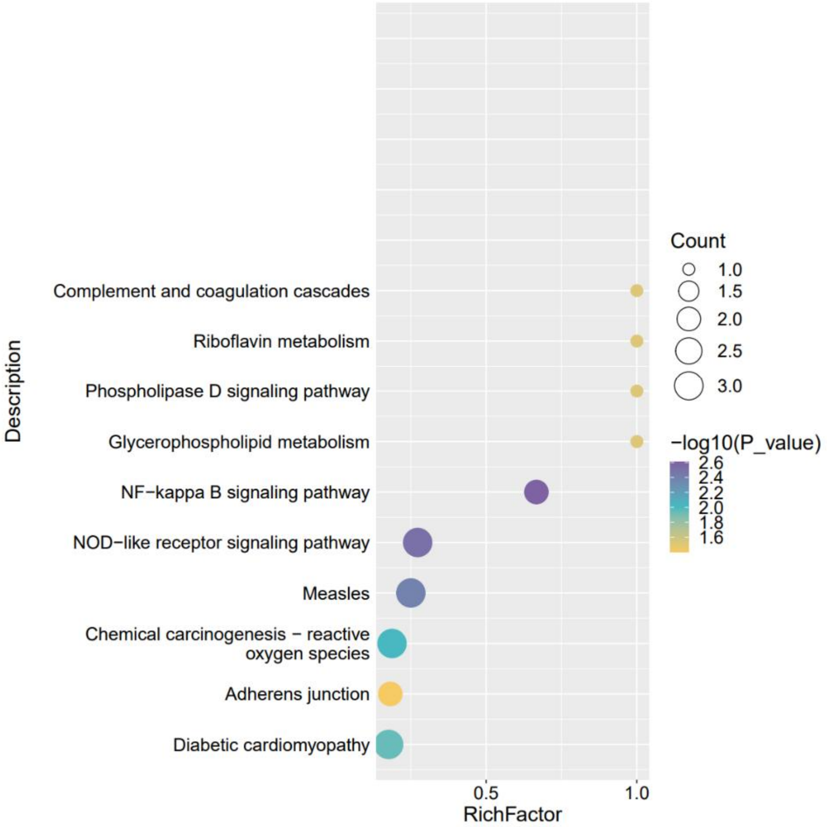

Figure S4. KEGG pathway enrichment of proteins interacting with RACK1.

Figure S5. Original Western Blot images.

S5a.

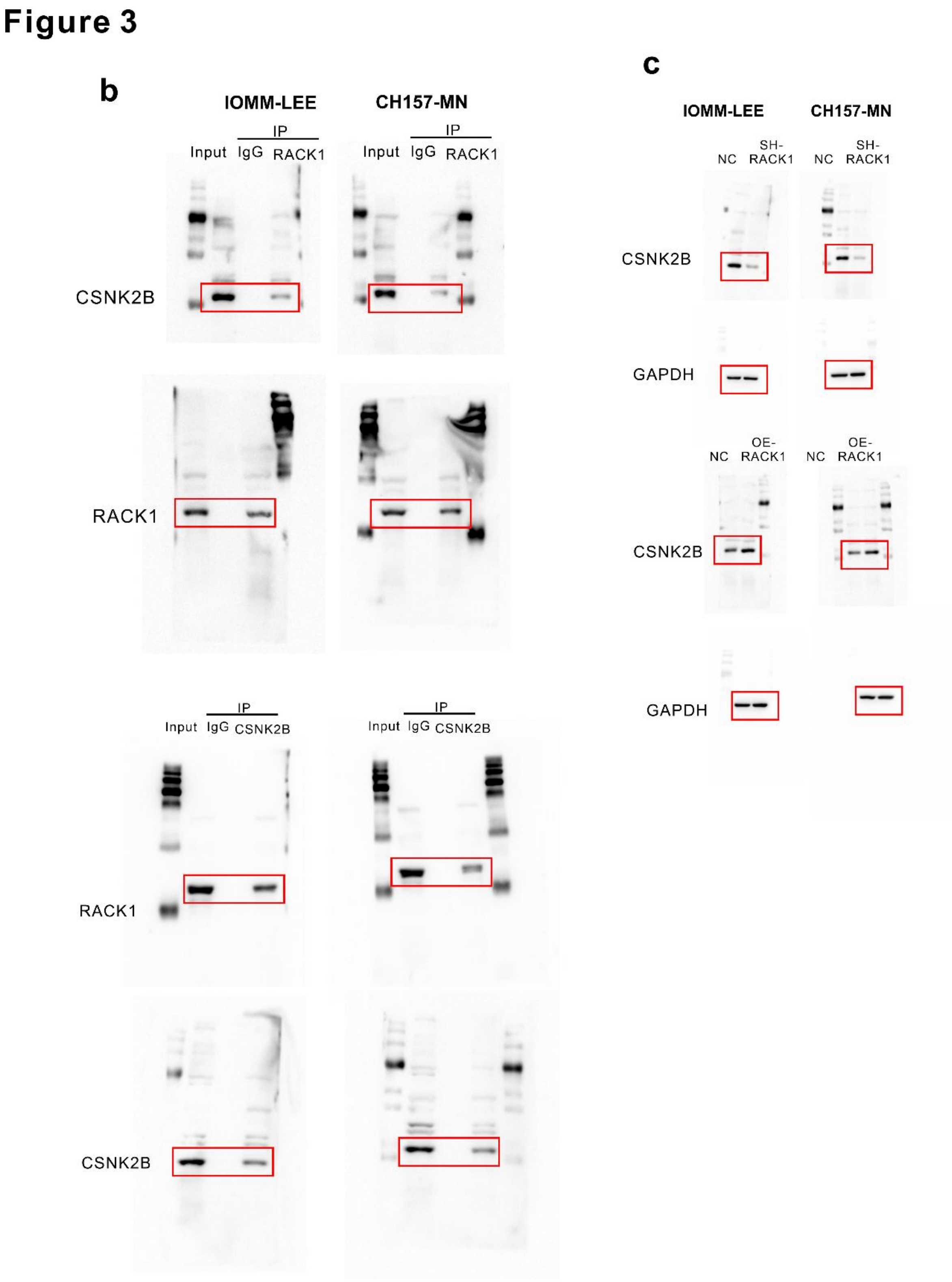

(b) Western blot of the whole-cell lysates (input) and Co-IP complex captured with anti-CSNK2B antibody and anti-RACK1 in IOMM-LEE and CH157-MN cells; (c) Western blot of IOMM-LEE and CH157-MN lysates whose RACK1 were knocked down or overexpressed.

S5b.

Figure 3

d

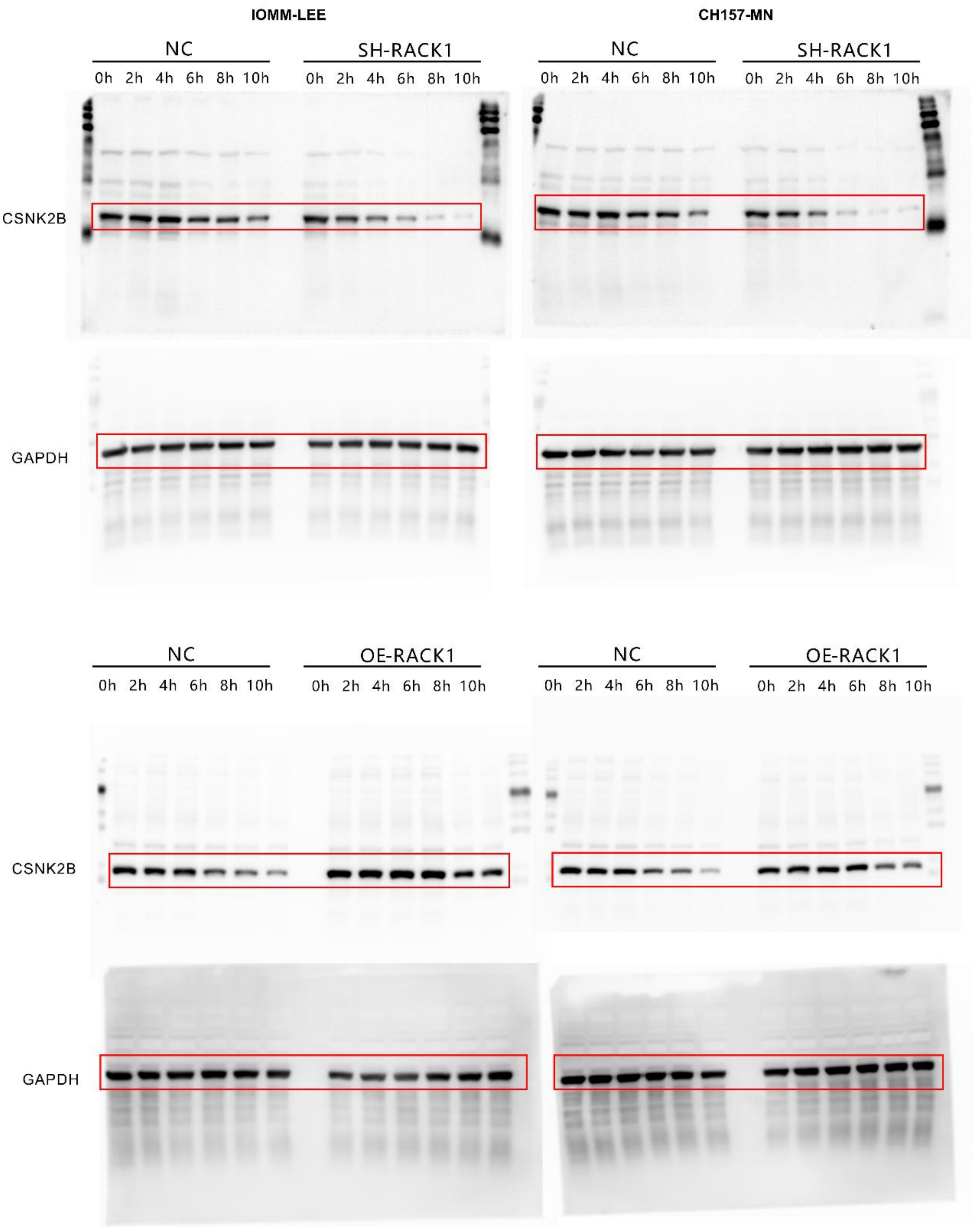

(d) Western blot of IOMM-LEE and CH157-MN lysates which were used with Cycloheximide (CHX) to inhibit protein synthesis.

S5c.

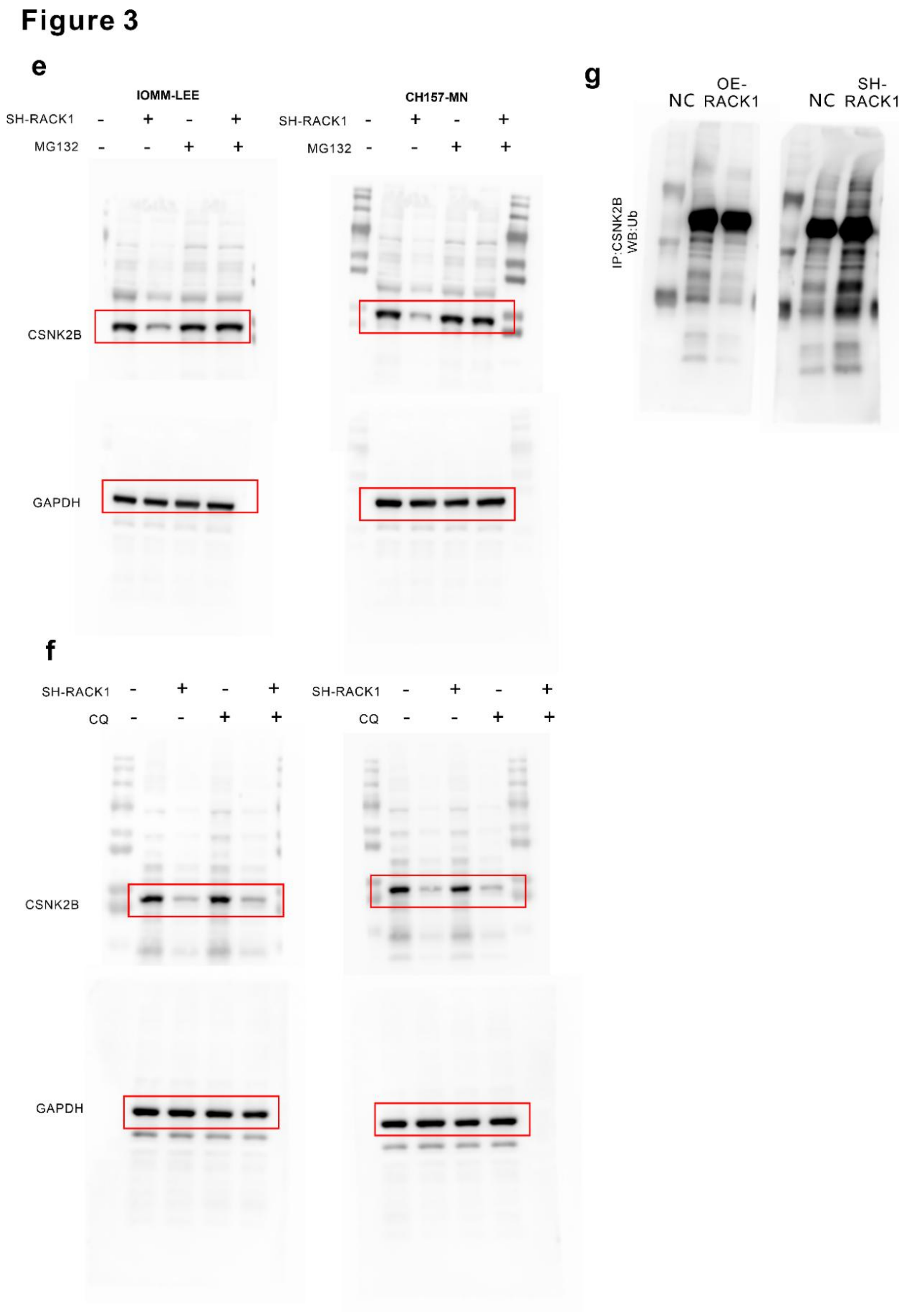

(e) Western blot of IOMM-LEE and CH157-MN lysates which were used with MG132; (f) Western blot of IOMM-LEE and CH157-MN lysates which were used with CQ; (g) Western blot of Co-IP complex captured with anti-CSNK2B antibody in IOMM-LEE cells to detect the ubiquitin levels.

S5d.

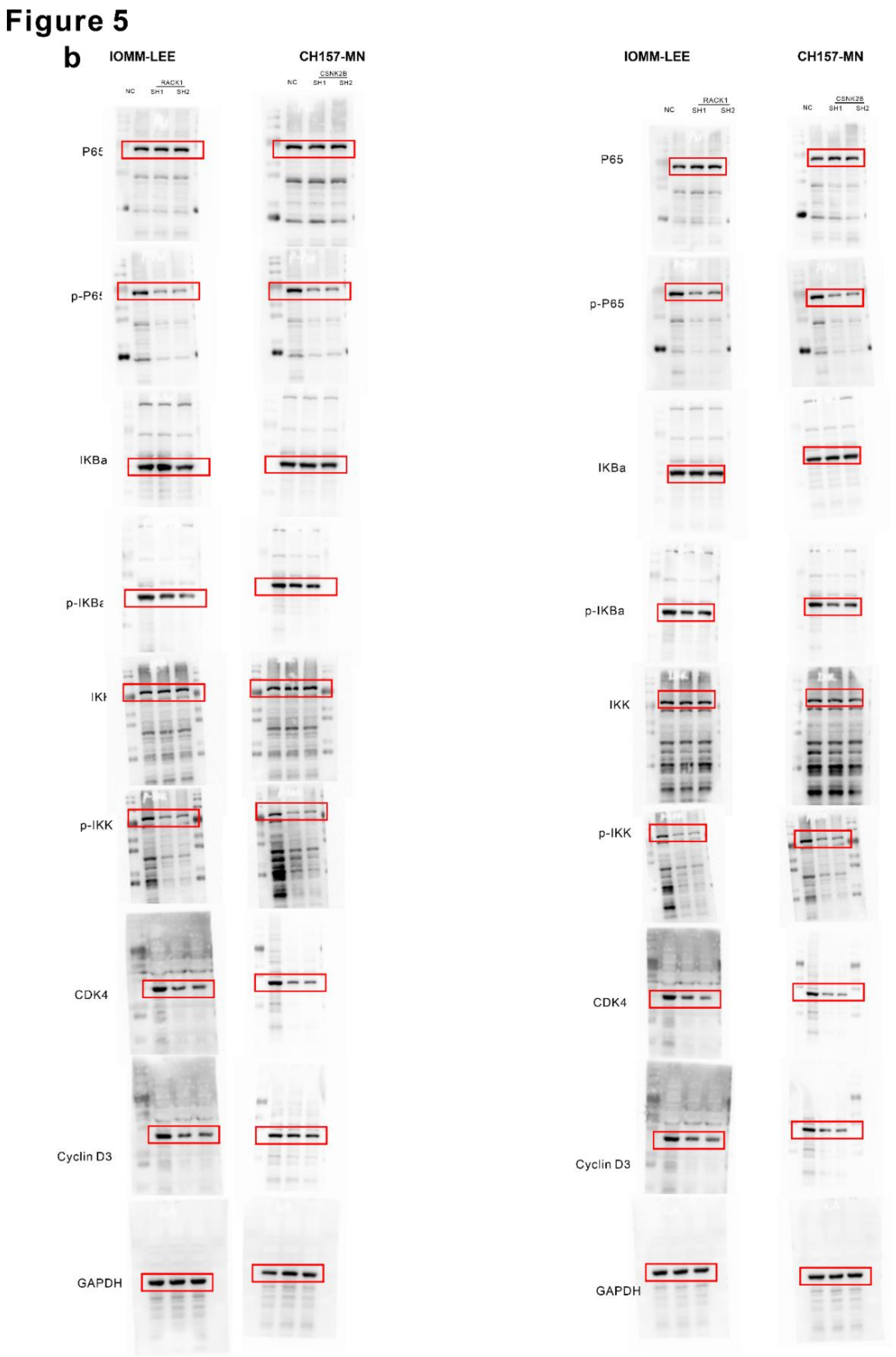

(b) Western blot of IOMM-LEE and CH157-MN lysates to detect the expression of NF-κB pathway protein.

S5e.

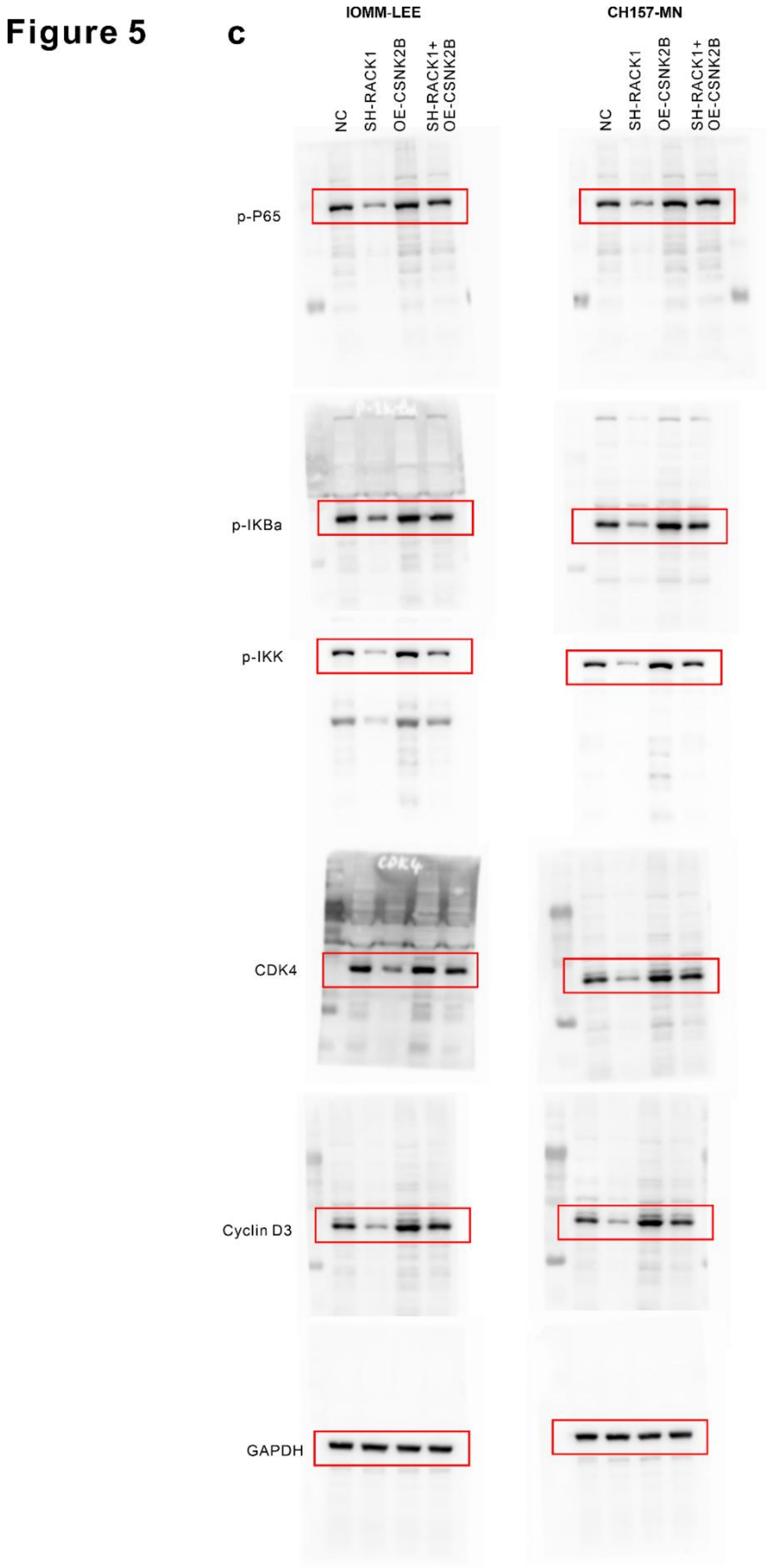

(c) Western blot of IOMM-LEE and CH157-MN lysates to detect the expression of NF-κB pathway protein.

S5f.

Figure 7

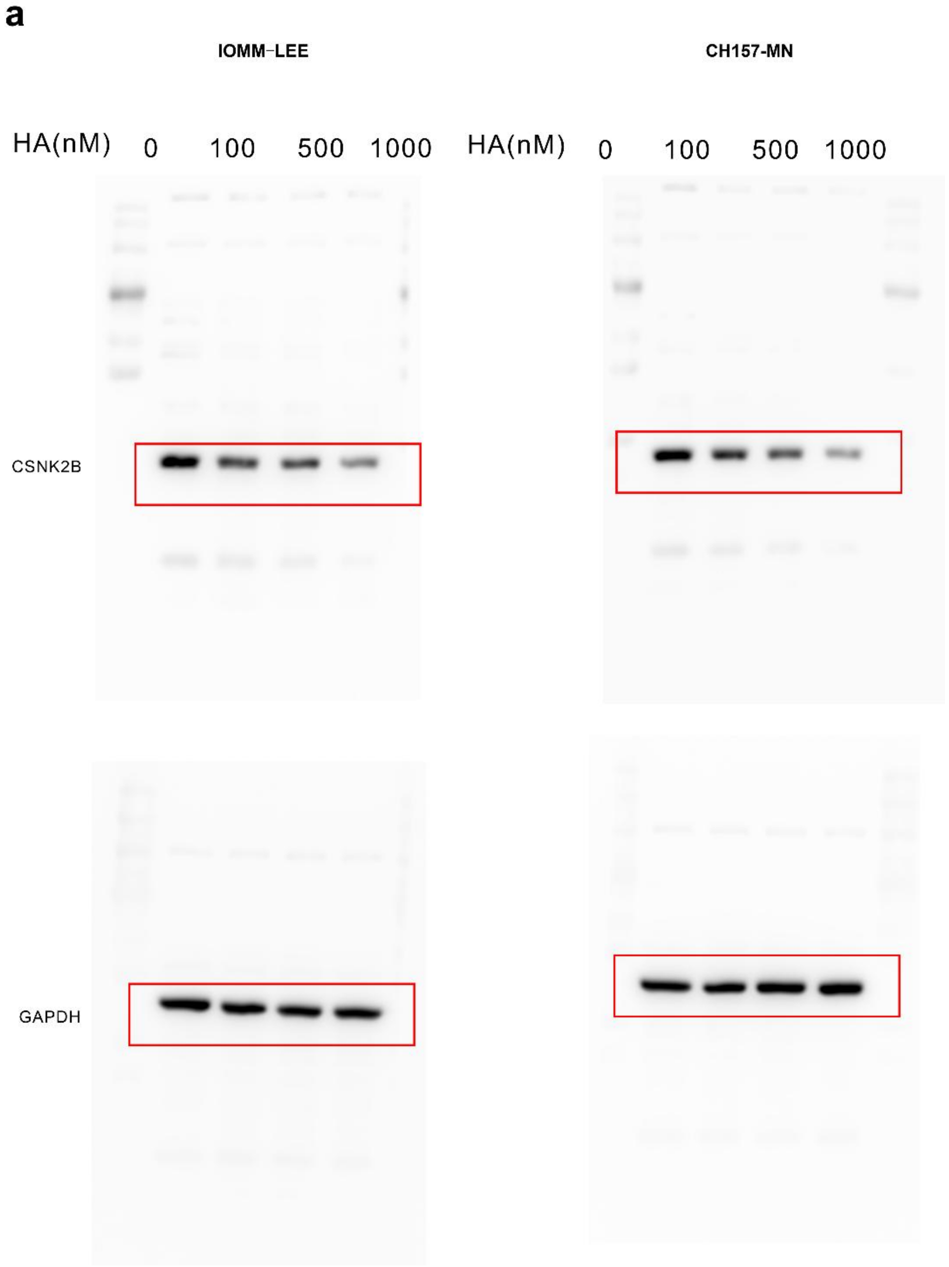

(a) Western blot of IOMM-LEE and CH157-MN lysates to detect the effect of HA on the expression of CSNK2B.
